# Echo and heart failure: when do people need an echo, and when do they need natriuretic peptides?

**DOI:** 10.1530/ERP-18-0004

**Published:** 2018-04-24

**Authors:** Daniel Modin, Ditte Madsen Andersen, Tor Biering-Sørensen

**Affiliations:** Department of Cardiology, Herlev & Gentofte Hospital, University of Copenhagen, Copenhagen, Denmark

**Keywords:** 2D echocardiography, 2D speckle-tracking echocardiography, heart failure, mechanics

## Abstract

Heart failure (HF) is a threat to public health. Heterogeneities in aetiology and phenotype complicate the diagnosis and management of HF. This is especially true when considering HF with preserved ejection fraction (HFpEF), which makes up 50% of HF cases. Natriuretic peptides may aid in establishing a working diagnosis in patients suspected of HF, but echocardiography remains the optimal choice for diagnosing HF. Echocardiography provides important prognostic information in both HF with reduced ejection fraction (HFrEF) and HFpEF. Traditionally, emphasis has been put on the left ventricular ejection fraction (LVEF). LVEF is useful for both diagnosis and prognosis in HFrEF. However, echocardiography offers more than this single parameter of systolic function, and for optimal risk assessment in HFrEF, an echocardiogram evaluating systolic, diastolic, left atrial and right ventricular function is beneficial. In this assessment echocardiographic modalities such as global longitudinal strain (GLS) by 2D speckle-tracking may be useful. LVEF offers little value in HFpEF and is neither helpful for diagnosis nor prognosis. Diastolic function quantified by E/e′ and systolic function determined by GLS offer prognostic insight in HFpEF. In HFpEF, other parameters of cardiac performance such as left atrial and right ventricular function evaluated by echocardiography also contribute with prognostic information. Hence, it is important to consider the entire echocardiogram and not focus solely on systolic function. Future research should focus on combining echocardiographic parameters into risk prediction models to adopt a more personalized approach to prognosis instead of identifying yet another echocardiographic biomarker.

## Introduction

Chronic heart failure (HF) represents a large societal burden of disease and has recently been characterized as an emerging epidemic (1). HF is associated with significant mortality and morbidity (1). Furthermore, healthcare expenditures are only expected to increase due to ageing of the population (2). As a result, strategies to prevent HF and improve the efficiency and quality of care are needed. HF is a clinical syndrome characterized by heterogeneities in both aetiology and phenotype, making management and intervention difficult. For example, it has become apparent that almost 50% of HF patients may have HF with preserved left ventricular (LV) ejection fraction (HFpEF) (3), a disease that represents a diagnostic, prognostic and therapeutic challenge. Echocardiography provides a large amount of detailed information regarding cardiac structure and function in an easily accessible and cost-effective manner and is currently recommended in the diagnostic workup of patients in whom HF cannot be ruled out clinically (4). Additionally, biomarkers such as type B natriuretic peptides (BNP) and N-terminal prohormone BNP (NT-proBNP) may aid in the diagnosis of HF (5). This review summarizes the important features, strengths and limitations of echocardiography and BNP HF with respect to diagnosis, prognosis and risk prediction. 

## Diagnosis of HF

The diagnosis of non-acute HF relies on the presence of HF-related symptoms and the subsequent quantification of cardiac dysfunction. Cardinal symptoms include but are not limited to dyspnoea, reduced exercise capacity and peripheral oedema. Comorbidities such as previous myocardial infarction increase the likelihood of a HF diagnosis (6). Many of these symptoms are non-specific for HF (7), especially in the setting of chronic lung disease (7). Therefore, in general, patients presenting with signs and/or symptoms of HF should undergo an echocardiogram to confirm HF diagnosis and to determine the underlying aetiology in order to guide treatment and management (4). In current guidelines, natriuretic peptides are recommended as an alternative initial screening protocol potentially capable of ruling out the presence of HF (4). BNP and NT-proBNP both display a questionable positive predictive value, but a very high negative predictive value with respect to ruling out the presence of HF with reduced ejection fraction (HFrEF) (8, 9). The high negative predictive value but low positive predictive value is likely due to contemporary cut-offs being very low. Current guidelines emphasize that patients suspected of HF with a BNP >35 pg/mL or a NT-proBNP >125 pg/mL must undergo echocardiography to confirm HF diagnosis (4) and that patients with values below the cut-offs are very unlikely to have HF. However, natriuretic peptide levels have been shown to increase significantly with age and female sex (10), and age-adjusted cut-offs may offer better discriminatory value in the elderly and avoid unnecessary echocardiograms (11). Also, in a recent study of patients with valvular disease and adverse cardiac remodelling but with normal LV systolic function, the majority of patients had normal BNP levels (12). More research is required to determine whether valvular disease may affect the diagnostic value of BNP. Still, echocardiography to confirm HF diagnosis is not recommended in contemporary guidelines if values of natriuretic peptides are below reported cut-offs (4). The rationale for this approach is sound, since a blood-based biomarker capable of ruling out HF allows for the prevention of unnecessary echocardiograms. Additionally, it allows the clinician to search for the true cause of the patient’s symptoms. However, it is known that values of NT-proBNP and BNP are lower in HFpEF than in HFrEF (13). 

**Figure 1:**
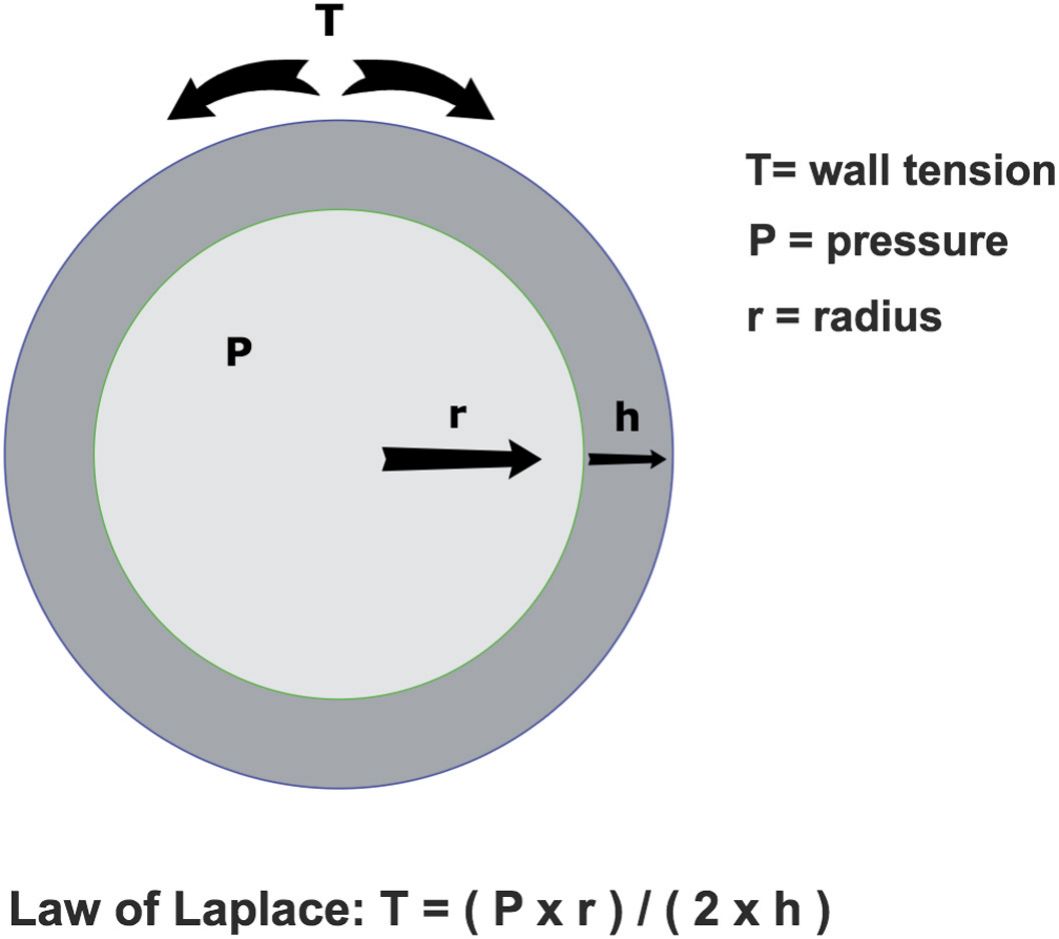
This figure shows the law of Laplace applied to a cross-sectional diagram of LV. The law of Laplace dictates that the LV wall tension is directly proportional to the product of the LV pressure and the LV radius. The LV wall tension is also inversely proportional to the LV wall thickness. LV, left ventricle.

Natriuretic peptides are secreted in response to myocardial wall stress. HFpEF is characterized by a small LV cavity and thickened LV walls (14). Since the law of Laplace (Fig. 1) dictates that LV wall stress is inversely proportional with LV wall thickness and directly proportional to LV radius, HFpEF does not elevate LV wall stress in the same way as seen in HFrEF (14, 15). Furthermore, it is known that values of natriuretic peptides are consistently lower in obese patients (16, 17, 18). Accordingly, it has been shown that obese HFpEF patients have lower levels of natriuretic peptides when compared to non-obese HFpEF patients (19). The mechanisms responsible for the lower levels of natriuretic peptides seen in obese HFpEF patients are currently unclear; however, it has been hypothesized that increased epicardial fat mass in obesity may subject the heart to an increased external pressure (19, 20). This increased external pressure then attenuates some of the intraventricular pressure that is believed to stimulate natriuretic peptide release, leading to reduced natriuretic peptide release (19, 20). When considering that almost 50% of all HF patients display a preserved EF phenotype (3) and that obesity is closely associated with HFpEF (21, 22), caution must be taken when excluding a HF diagnosis on the basis of a BNP measurement of <35 pg/mL or a NT-proBNP <125 pg/mL as recommended in current guidelines (4). The high prevalence of morbid obesity in HFpEF decreases the diagnostic value of natriuretic peptides, and it also complicates the estimation of jugular venous pressure and other diagnostic signs such as oedema. It should be noted that common cardiovascular medications such as angiotensin-converting enzyme inhibitors, angiotensin II receptor antagonists and diuretics may reduce circulating levels of BNP (23, 24, 25, 26). Therefore, low BNP values must be interpreted with care in patients already taking these medications. Hence, diagnosing HFpEF remains challenging, and the clinician must remain vigilant. The possibility of HFpEF despite near-normal levels of natriuretic peptides especially in the setting of morbidly obese patients must still be considered and, if suspected, followed up by echocardiography.

HFrEF is easily diagnosed by echocardiography. The diagnosis of HFrEF, is by definition HF symptoms and left ventricular ejection fraction (LVEF) <40%, usually quantified by the Simpson biplane method (4). 

HFpEF is more difficult to diagnose and the diagnosis includes, in addition to HF symptoms and a LVEF ≥50%, structural or functional signs of diastolic dysfunction or LV hypertrophy. These include either left atrial dilation (left atrial volume index ≥34 m/m^2^), LV hypertrophy (left ventricular mass index ≥115 g/m^2^ for men and ≥95 g/m^2^ for women) or an E/e′ ≥13 (4). As has been shown for the NT-proBNP and BNP cut-offs values, these criteria perform mediocre at best in diagnosing HFpEF (27). However, a recent study by Obokata *et al*. suggests that adding E/e′ measured during exercise to current guidelines may increase the sensitivity and negative predictive value for ruling out HFpEF (27). Importantly, it must be noted that Obokata *et al*. used E/e′ cut-offs from the American Society of Echocardiography/European Associaton of Cardiovascular Imaging (ASE/EACV) guidelines for assessment of diastolic dysfunction (27, 28). Therefore, the E/e′ cut-off for diastolic dysfunction measured during exercise differs with the measurement position of e′. When using only e′ measured in the lateral mitral annulus, a cut-off of E/e′ >15 is employed, while an E/e′ cut-off value of E/e′ >14 is used when e′ is averaged from both the septal and lateral mitral annular position (27, 28). Hence, when using exercise E/e′ in HFpEF diagnostics the e′ measurement position must be accounted for. New techniques such as myocardial strain deformation imaging by 2D speckle-tracking (2DS) have been shown to detect impaired systolic function in HFpEF despite normal LVEF (29). Furthermore, 2DS at rest has been demonstrated to identify patients with an increasing filling pressure during exercise among patients with unexplained dyspnoea and a normal LVEF (30). Hence, in the time to come, deformation imaging by 2DS may ease the diagnosis of HFpEF.

Finally, a new group of patients has been introduced in the latest 2016 European Society of Cardiology (ESC) HF guidelines. This patient group has been termed ‘heart failure with mid-range ejection fraction’ (HFmrEF), and comprises patients with heart failure symptoms, a LVEF of 40–49%, elevated levels of natriuretic peptides and either relevant structural heart disease (LV hypertrophy or left atrial (LA) enlargement) or diastolic dysfunction (4). According to the ESC, introducing this patient group as an entity independent of HFpEF and HFrEF was done to ‘stimulate research into the underlying characteristics, pathophysiology and treatment of this group of patients’ (4). Patients with HFmrEF are estimated to comprise 10–20% of all HF patients and currently occupies a ‘grey zone’ in the HF literature (31). In the cardiovascular health study, the mortality of HFmrEF patients was intermediate between HFrEF and HFpEF (32). It is interesting that some of the diagnostic criteria for HFmrEF are identical to those of HFpEF (signs of relevant structural heart disease or diastolic dysfunction) (4). This in accordance with recent evidence suggesting that HFmrEF may constitute a subset of HFpEF patients who are more affected by coronary artery disease (31). Coronary artery disease in HFpEF is associated with worse outcome and greater deterioration in LVEF, and some HFmrEF patients may therefore be HFpEF patients who may be progressing to HFrEF (33). However, large gaps in evidence regarding HFmrEF exist, and the introduction of HFmrEF as a diagnostic entity independent of HFrEF and HFpEF in current HF guidelines is likely to spur much-needed future research into this conundrum. 

## Prognosis and risk prediction in HF

In the current guidelines, echocardiography is recommended in the diagnostic workup of suspected HF patients in order to establish a diagnosis of either HFrEF or HFpEF (4). Echocardiography is also recommended in HFrEF patients to assess LVEF in order to guide evidence-based pharmacological treatment and device therapy (implantable cardioverter defibrillator (ICD) and cardiac resynchronization therapy (CRT)) and to quantify valvular disease (4). In addition, echocardiographic assessment of HF patients provides very important prognostic information. This is essential in helping patients, families and clinicians decide on appropriate type and timing of therapy. 

HF patients who have undergone echocardiographic examination show better survival rates due to intensified medical treatment and intervention (34). Many echocardiographic markers have displayed prognostic value in HF (Table 1), and echocardiography is vital in the risk stratification of HF patients (35). LVEF determined by echocardiography is widely used in clinical practice and currently guides both diagnosis and therapy in HF (4). However, new and promising methods such as strain imaging by 2DS and tissue Doppler imaging (TDI) have emerged. Particularly, strain imaging has proven beneficial in detecting impaired systolic function in HFpEF despite normal LVEF values. The following sections will discuss risk prediction in HF including new methods such as 2DS and TDI.

**Table 1:** The results of selected studies that have identified echocardiographic prognostic markers in both HFrEF and HFpEF.

Study (year)	Echo parameter	Outcome	*N*	Follow-up	Comment
**HFrEF**					
Systolic function					
Curtis *et al*. 2003 (37)	LVEF	All-cause mortality	7788	37 months (mean)	
Pocock *et al*. 2006 (38)	LVEF	All-cause mortality, cardiac death and HF hospitalization (composite)	7599	38 months (median)	
Sengeløv *et al*. 2015 (39)	GLS	All-cause mortality	1065	40 months (median)	Superior to LVEF
Hasselberg *et al*. 2015 (40)	GLS	Exercise capacity	63	N/A	Superior to LVEF
Risum *et al*. 2013 (44)	LV dyssynchrony by TDI	CRT response (all-cause mortality, cardiac transplantation or LVAD) (composite)	131	47 months (truncated)	
Haugaa *et al*. 2012 (114)	LV mechanical dispersion*	Ventricular fibrillation or tachycardia (composite)	569	30 months (median)	Following myocardial infarction
Biering-Sørensen *et al*. 2017 (48)	LV strain the inferior wall	Ventricular fibrillation or tachycardia (composite)	1064	35 months (median)	MADIT-CRT sub-study
Biering-Sørensen *et al*. 2016 (49)	Inferior wall late diastolic velocity (a′) by TDI	Ventricular fibrillation or tachycardia or cardiac death(composite)	151	28 months (median)	
Modin *et al*. 2017 (53)	GLS corrected by RR-interval	All-cause mortality	151	32 months (median)	HFrEF with atrial fibrillation during examination
Diastolic and RV function					
Pinamonti *et al*. 1993 (54)	Restrictive filling pattern by E/A and DT	All-cause mortality or cardiac transplantation (composite)	79	22 months	
Xie *et al*. 1994 (55)	Restrictive filling pattern by E/A and DT	Cardiac death	100	16 months (mean)	
Acil *et al*. 2005 (57)	E/e′	Cardiac death, cardiac transplantation or HF hospitalization (composite)	132	7.5 months (mean)	
Rossi *et al*. 2009 (58)	LA area	All-cause mortality or HF hospitalization (composite)	1157	N/A	Meta-analysis of 18 prospective studies
Hsiao & Chiou 2013 (59)	LA expansion index	All-cause mortality and HF admission (composite)	1735	31 months (median)	Dyspnoea patients
Ghio *et al*. 2001 (60)	RV ejection fraction	All-cause mortality or Cardiac transplant (composite)	377	17 months (median)	
**HFpEF**					
Systolic function					
Shah *et al*. 2015 (89)	GLS	Cardiovascular death, HF hospitalization or aborted cardiac arrest (composite)	447	31 months (median)	TOPCAT sub-study
Huang *et al*. 2017 (90)	GLS	All-cause mortality or HF hospitalization (composite)	54	At least 3 years	
Biering-Sørensen *et al*. 2017 (30)	GLS	Exercise-induced rise in pulmonary arterial wedge pressure	85	N/A	Unexplained dyspnoea patients
Hasselberg *et al*. 2015 (40)	GLS	Exercise capacity	37	N/A	
Wang *et al*. 2015 (92)	GLS during exercise	All-cause mortality or HF hospitalization (composite)	80	36 months	
Other parameters					
Okura *et al*. 2009 (96)	E/e′	All-cause mortality or HF hospitalization (composite)	50	19 months (mean)	
Santos *et al*. 2016 (105)	LA strain	Cardiovascular death, HF hospitalization or aborted cardiac arrest (composite)	357	31 months (mean)	TOPCAT sub-study
Melenovsky *et al*. 2015 (115)	LA emptying fraction	All-cause mortality	101	12 months (median)	
Lam *et al*. 2009 (103)	Tricuspid regurtitant velocity	All-cause mortality	244	36 months (median)	
Melenovsky *et al*. 2014 (116)	RV fractional area change	All-cause mortality	96	18 months (median)	
Mohammed *et al*. 2014 (105)	TAPSE	All-cause mortality, cardiovascular mortality and HF hospitalization (not composite)	562	55 months	

CRT, cardiac resynchronization therapy; DT, deceleration time of the E-wave; GLS, global longitudinal strain; HF, heart failure; LA, left atrial; LV, left ventricular; LVEF, left ventricular ejection fraction; RV, right ventricle; TAPSE, tricuspid annular plane systolic excursion; TDI, tissue Doppler imaging.

## Risk prediction in HFrEF

Echocardiography is very valuable in the risk stratification of HFrEF patients. In 1962, Folse and Braunwald published results describing how to measure the ‘fraction of LV volume ejected per beat’ (36). This study marked an era spanning decades in which LVEF was the single most important metric in echocardiography, and particularly so in HFrEF. We now know that both anatomical structure and cardiac function offer prognostic insight in HFrEF and for that reason, it is important to do a comprehensive echocardiogram. This includes evaluating both LV systolic and diastolic function in addition to right ventricular and LA function. During the past 10–15 years, it has become increasingly apparent that advanced methods such as 2DS and TDI provide valuable insight into the prognosis and natural history of HF. This is acknowledged in current HF guidelines, since advanced methods should be considered for the detection of subclinical cardiac dysfunction in individuals at high risk of developing HF (4). LVEF still remains an important measurement in HFrEF (4), but as we now know, echocardiography has more information to offer.

### Systolic function and prognosis in HFrEF

Reduced systolic function confers an adverse prognosis in HFrEF. LVEF remains the most widely used echocardiographic parameter for quantification of systolic function and is an established predictor of mortality in HFrEF (37, 38). However, LVEF relies on geometric assumptions and may therefore not reflect actual LV deformation. Recently, global longitudinal strain (GLS) has been demonstrated as a superior predictor of mortality in HFrEF when compared to LVEF (39). GLS is also superior to LVEF in predicting reduced exercise capacity in HFrEF (40). This suggests that GLS may be able to quantify the extent of systolic dysfunction in HFrEF more accurately and that it may be a superior prognostic factor to LVEF. 

Two major causes of death in HFrEF are cardiac pump failure and sudden death from malignant ventricular arrhythmias. Device-based therapy such as CRT and CRT-ICD has been shown to reduce mortality and rehospitalization rates and improve prognosis in selected subsets of HFrEF patients (41). However, this therapy is very costly, and in several trials, it has been noted that approximately 1/3 of patients under current HFrEF selection criteria do not benefit clinically or hemodynamically (i.e. with an increased LVEF) from this treatment (42). Furthermore, the heterogeneous pathophysiology underlying HFrEF complicates the selection of patients. Current selection criteria are LVEF ≤35%, a wide QRS complex (≥150 ms) and symptomatic HF (43). Previous attempts to use parameters derived from echocardiography for the selection of CRT candidates have failed. However, mechanical dyssynchrony assessed by TDI has been associated with long-term survival in CRT patients (44), and measures of LV dyssynchrony based on longitudinal strain imaging appear to be strong prognostic factors of malignant arrhythmias in HFrEF (45, 46). Thus, these methods are promising for improving the selection of HFrEF patients for CRT and CRT-ICD. 

Beside the geometric assumptions, another significant disadvantage of the LVEF is the lack of ability to quantify regional myocardial function. In mammalian hearts, reentry circuits arise when a wave of electrical activation abnormally reenters the myocardium instead of propagating normally throughout the cardiac tissue to die out in its periphery. Reentry plays a significant role in the pathophysiology underlying life-threatening ventricular arrhythmias such as ventricular tachycardia (VT) or ventricular fibrillation (VF) (47). Arrhythmias sustained by reentry mechanisms rely primarily on heterogeneities in cardiac structure and function. Localized areas of abnormal cardiac anatomical structure (such as scarring/fibrosis) or electrophysiological properties (such as subclinical ischaemia) may contribute to arrhythmogenesis. These localized areas can be missed by dilution with global measures of cardiac function such as the LVEF. In HFrEF patients receiving ICD-CRT therapy from the Multicenter Automatic Defibrillator Implantation Trial with Cardiac Resynchronization Therapy (MADIT-CRT) trial, only reduced peak longitudinal strain in the inferior wall predicted VT/VF (48). This prognostic value was incremental to clinical and echocardiographic parameters (including GLS). Furthermore, in HFrEF patients with ischaemic aetiology receiving ICD therapy, only the late diastolic velocity (a′) measured by TDI in the inferior wall predicted a combined outcome of VT/VF and cardiovascular death (49). These results show that regional function is important in the diagnosis, treatment and prognosis of HFrEF.

It is now apparent that quantification of systolic function offers much prognostic value in HFrEF. However, the high prevalence of atrial fibrillation (AF) in HFrEF represents a challenge to current echocardiographic methods. In AF rhythm, the varying RR-interval and changing loading conditions impairs systolic measurements and thus the usefulness of these to predict outcome (50). Hence, AF patients are often excluded from echocardiographic studies. This is an issue when considering the very high prevalence of AF in HFrEF (51). A novel method of correcting GLS values by the RR-interval has been suggested (52) and has recently been demonstrated to be a superior prognostic marker to LVEF in HFrEF patients with AF during examination (53). This method may allow risk stratification of HFrEF patients despite AF rhythm. 

### Comprehensive cardiac assessment and prognosis in HFrEF

In HFrEF, much emphasis is put on the quantification of systolic function. Other aspects of cardiac structure and function also contribute with prognostic value. The quantification of LV filling pressure holds prognostic value in HFrEF: A restricted filling pattern by Doppler echocardiography as determined by E/A ratio and deceleration time of the E-wave is highly prognostic in HFrEF (54, 55). The ratio of transmitral early LV filling velocity to early diastolic TDI velocity of the mitral annulus (E/e′) is a measure of LV filling pressure and diastolic function. E/e′ is an independent predictor of mortality and hospitalization in HFrEF (56, 57). LA volume and function, important measures of diastolic function and markers of LV filling pressure, also contribute with independent prognostic value in HFrEF. LA size has been demonstrated as a powerful predictor of mortality and hospitalization in a meta-analysis of 18 studies of HFrEF patients (58). Particularly, the quantification of LA function through the LA emptying fraction and the LA expansion index seems promising. In a study of 1735 dyspnoea patients, LA expansion index was superior to LA volume in predicting mortality and hospitalization for HF (59). Thus, information about LV diastolic function provides much prognostic information in HFrEF. 

The left side of the heart is not the sole contributor to risk stratification in HFrEF. The right ventricle (RV) holds significant prognostic value in HFrEF. A common misconception – it is thought that RV systolic function is exclusively determined by the afterload posed by decreasing LV function. However, RV ejection fraction and pulmonary artery systolic pressure both independently predict outcome in HFrEF (60). Thus, the prognostic value of RV systolic function is independent of RV afterload secondary to LV dysfunction and decreased RV systolic function likely marks a stage of advanced disease in which RV compensation is no longer possible.

**Figure 2:**
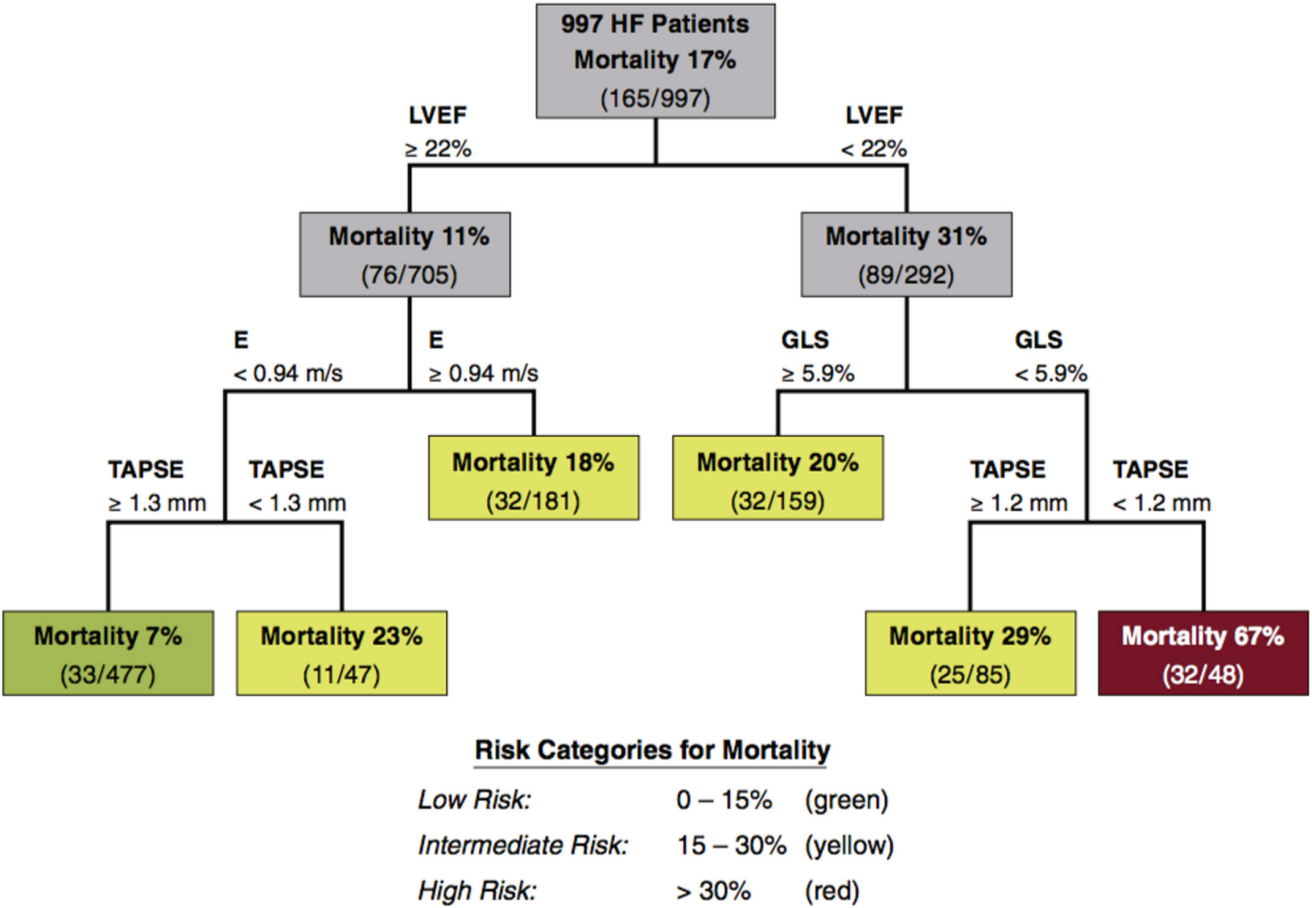
A risk stratification tree obtained by CART analysis. A CART analysis includes many echocardiographic parameters to determine the most important predictors of mortality in HFrEF patients. The analysis selected LVEF, GLS, peak early diastolic filling velocity (E) and TAPSE as the most important predictors of mortality in HFrEF and combined them into a binary risk assessment scheme. CART, classification and regression tree analysis; GLS, global longitudinal strain; HF, heart failure; HFrEF, HF with reduced ejection fraction; LVEF, left ventricular ejection fraction; TAPSE, tricuspid annular plane systolic excursion. Reprinted from *JACC: Cardiovascular Imaging*, Vol 8, Sengeløv M, Jørgensen PG, Jensen JS, Bruun NE, Olsen FJ, Fritz-Hansen T, Nochioka K & Biering-Sørensen T, Global longitudinal strain is a superior predictor of all-cause mortality in heart failure with reduced ejection fraction, Pages 1351–1359, Copyright (2015), with permission from Elsevier .

The aforementioned are in no way an exhaustive list of every echocardiographic marker or parameter that holds prognostic value in HFrEF. There are many more, such as the quantification of chamber geometry and valvular disease and indices of LV mass and hypertrophy. It serves to illustrate that the prognostic value of echocardiography in HFrEF goes far beyond the LVEF. Even though GLS shows promise as a universal marker of cardiac function, no single prognostic factor is sufficient for risk assessment in HFrEF. This was elegantly demonstrated by Sengeløv *et al*. in a study of 1065 HFrEF patients (39). In this study, GLS was the best prognostic factor out of all echocardiographic parameters determined by multivariable Cox regression and univariable C-statistics. Authors also performed a classification and regression tree (CART) analysis of echocardiographic parameters included in the study. CART is a statistical technique used to determine the best binary risk assessment scheme with respect to prediction of an outcome (61). Through the CART analysis, when considering all echocardiographic parameters included in their study, Sengeløv *et al*. found that LVEF, GLS, E and tricuspid annular plane systolic excursion (TAPSE) were important in the risk stratification of their HFrEF cohort (39) (Fig. 2). These results emphasize the need to evaluate both systolic, diastolic and RV function when predicting risk in HFrEF (Table 1). They also serve to emphasize that no single prognostic marker is sufficient to predict prognosis in HFrEF and that the results of different echocardiographic parameters must be interpreted together and not as a collection of single markers of risk, independent of each other Table 1 provides a selection of the many echocardiographic predictors of outcome in HFrEF (Table 1).

### B-type natriuretic peptides and prognosis in HFrEF

The measurement of BNP to aid in risk stratification of chronic HF patients is recommended in current guidelines (recommendation Class 1A). Hence, BNP assessment is useful for determining risk of adverse outcome in chronic HF (62). BNP predicts all-cause mortality (63, 64) and sudden death in HFrEF (65). Furthermore, changes in BNP over a 6-month period have been shown to predict adverse outcome independently of baseline BNP levels (66). Thus, BNP levels offer easily accessible prognostic value in HFrEF and may be helpful in management and monitoring of HFrEF. 

## Risk prediction in HF with preserved ejection fraction

HFpEF currently represents a substantial clinical conundrum. Although spironolactone has been shown to reduce heart failure hospitalization rates in HFpEF (67), no therapeutic treatment has been shown to consistently improve survival (4). When considering that up to around 50% of HF patients may have HFpEF (3), this lack of effective therapeutic treatment represents a large unmet need in current cardiology practice. In order to properly orchestrate trials and to guide clinical decision making, thorough and accurate risk prediction is vital. 

### Impaired systolic function in HFpEF despite preserved LVEF

HFpEF was originally thought to result from diastolic dysfunction. This was based on invasive hemodynamic studies displaying increased LV stiffness, impaired diastolic relaxation and increased filling pressure in HFpEF (68, 69). However, even though LVEF may be preserved in HFpEF, systolic function is still abnormal. The LV contraction comprises longitudinal shortening, circumferential shortening and radial thickening. Both mitral annular plane longitudinal descent and velocity are impaired in HFpEF indicating decreased longitudinal function (70). GLS quantifies LV wall shortening during the cardiac cycle and particularly reflects longitudinal function (71). Accordingly, GLS has been shown to be impaired in HFpEF (29). Thus, despite a normal LVEF, systolic function is indeed abnormal in HFpEF.

**Figure 3:**
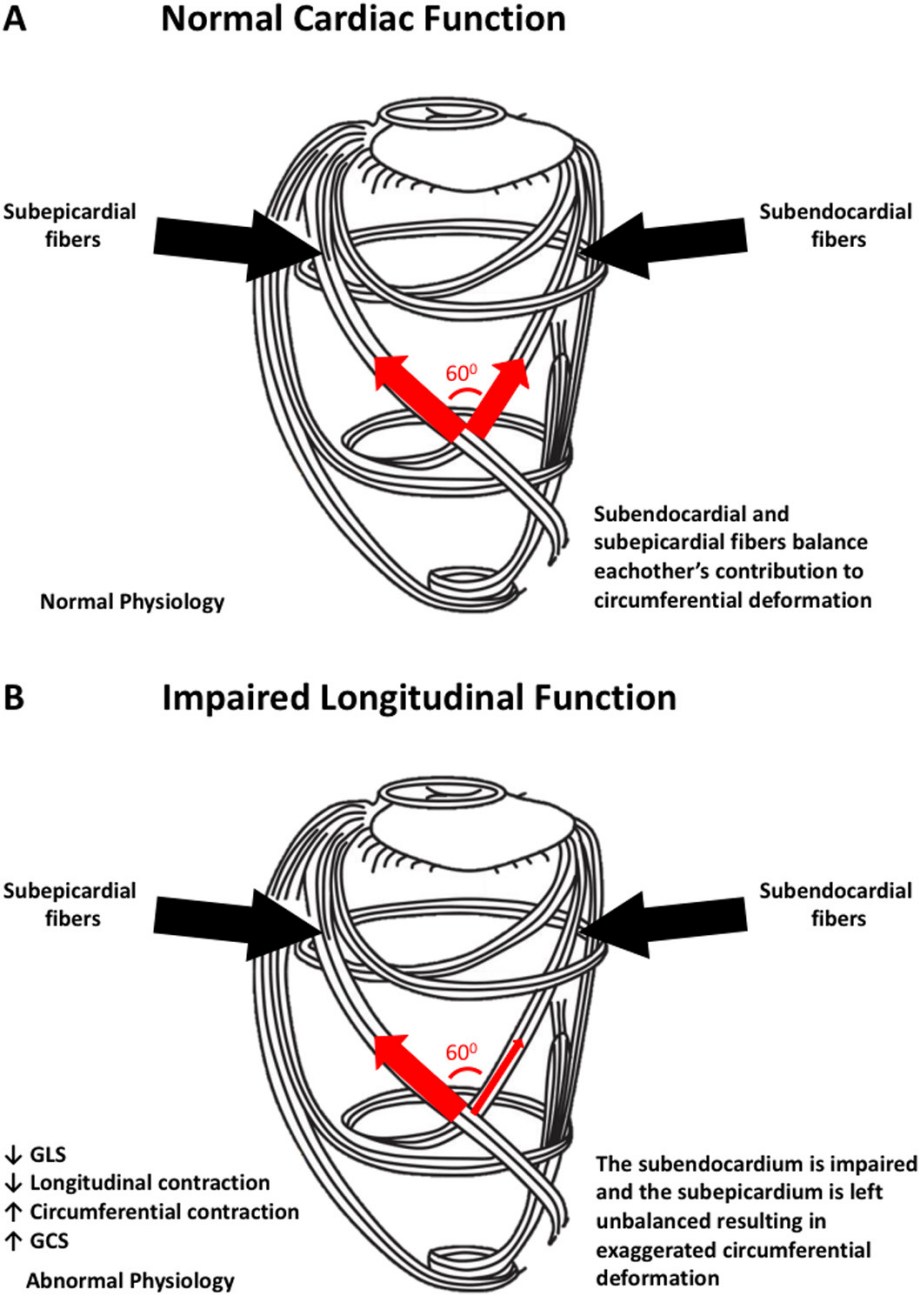
This figure depicts the myocardial fibre orientation of the left ventricular wall and their directions of contraction. In the subepicardium, myocardial fibres are oriented in a left-handed helix, while they run in a right-handed helix in the subendocardium. The cardiac midwall comprises circumferentially oriented fibres. (A) In the normal heart, the subepicardial left-handed helical fibres are balanced by the subendocardial right-handed helical fibres and longitudinal function is normal. (B) The subendocardial fibres are most susceptible to dysfunction from hypertension, increasing age, diabetes and other cardiovascular risk factors. When subendocardial function is lost, longitudinal contraction is impaired and the subepicardial fibres are left unbalanced. This results in decreased GLS and exaggerated circumferential contraction and GCS. This pattern of contraction is common in the presence of cardiovascular risk factors such as hypertension, increasing age and diabetes. GLS, global longitudinal strain; GCS, global circumferential strain. Adapted, under the terms of the original Creative Commons Attribution licence, from Nakatani 2011 (113).

**Figure 4:**
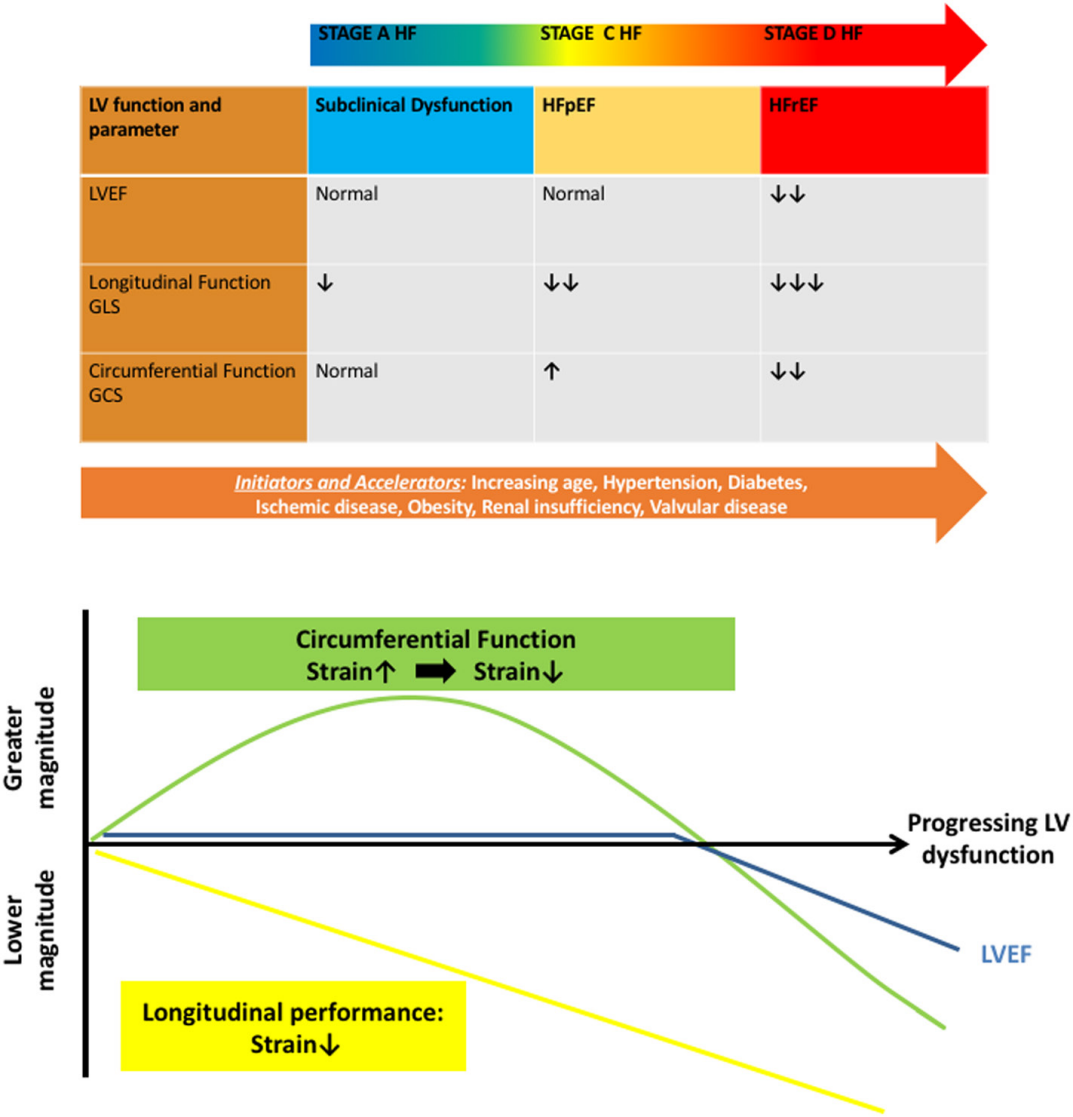
A model of progressive abnormalities in left ventricular function in heart failure across LVEF spectrum. Subclinical myocardial dysfunction triggered by cardiovascular risk factors such as age, hypertension and diabetes may present as depressed longitudinal deformation and decreased GLS but increased circumferential deformation and GCS. Progression is characterized by continuous impairment in longitudinal deformation. LVEF decreases at a point when circumferential function also starts to decline. GLS, global longitudinal strain; GCS, global circumferential strain; LVEF, LV ejection fraction; HFpEF, heart failure with preserved ejection fraction; HFrEF, heart failure with reduced ejection fraction.

How LVEF can be preserved, despite the presence of systolic impairment in HFpEF, is not entirely clear. An analysis of the LV fibre and contraction pattern may offer some insight into the conundrum that is HFpEF. The LV muscular wall comprises three overall compartments: the subendocardium, the midmyocardium and the subepicardium (72). Circumferential fibres occupy the midmyocardium and produce primarily circumferential shortening, while longitudinal fibres in the subendocardium and subepicardium form a right-handed and left-handed helix, respectively (72). Thus, the subendocardial and subepicardial fibres form two oppositely directed spirals, with a net difference in angulation between these two spirals ranging from +60° to −60° (Fig. 3) (73). As a result, the circumferential components of subendocardial and subepicardial fibre contraction balance each other out and produce little net circumferential shortening in the normal heart (Fig. 3). The subendocardial fibres appear to be the most susceptible to injury (74, 75). Impairments in subendocardial fibre function lead to decreased right-handed helix shortening and thus reduced longitudinal function. Additionally, impairments in subendocardial fibre function may leave the left-handed helix shortening by subepicardial fibres unbalanced, potentially resulting in increased circumferential shortening (Fig. 3) (29, 76, 77, 78). This mechanism of exaggerated circumferential shortening by subendocardial fibre dysfunction may explain a distinct pattern of contraction observed in many conditions of subclinical LV dysfunction predisposing to HFpEF (Fig. 4). In increasing age (79), hypertension (80), diabetes mellitus (81) and obesity (82), GLS is reduced, reflecting subendocardial fibre dysfunction; yet, LVEF is preserved. Accordingly, in many of these conditions, circumferential shortening appears to be preserved or increased (83, 84, 85, 86). This may be extended to explain the decreased longitudinal yet preserved or exaggerated circumferential function seen in HFpEF (85, 87) and may also explain how LVEF can be preserved yet systolic function impaired in HFpEF (Fig. 4). These considerations serve to emphasize the limitations of LVEF as the sole marker of LV systolic function.

### Systolic function and prognosis in HFpEF

In the Candesartan in heart failure - assessment of mortality and morbidity (CHARM) trials, which studied 7599 HF patients with a broad spectrum of LVEFs, LVEF did not accurately discriminate risk of cardiovascular outcome in patients with an LVEF >45% (88). A similar relationship between LVEF and mortality was found in the Digitalis investigation group (DIG) trial of 7788 HF patients (37). This suggests that LVEF does not accurately quantify risk of adverse outcome in HFpEF. However, as discussed previously, this does not mean that systolic function is normal in HFpEF, since longitudinal function determined by GLS has been shown to be impaired (29). In the recent years, GLS has emerged as a powerful prognostic factor of cardiovascular death and hospitalization in HFpEF (89). GLS was also a strong prognostic marker of mortality in index hospitalized HFpEF patients (90). Furthermore, GLS predicts reduced exercise capacity in HFpEF (40). Exercise capacity is a strong prognostic parameter in HFpEF (91). GLS measured during bicycle ergometer testing has also been demonstrated as a strong prognostic marker in HFpEF (92). Thus, GLS shows great promise in risk stratification of HFpEF patients (Table 1). In the future, GLS may become valuable in guiding patient selection for HFpEF trials and for directing therapeutic treatment. 

### Other echocardiographic parameters with prognostic value in HFpEF 

HFpEF is usually characterized by a small LV cavity, hypertrophied LV walls and severe diastolic dysfunction (93). Naturally, LV filling pressures are elevated in HFpEF (94) and LV compliance and relaxation is impaired, particular so during exercise (95). E/e′ is an estimate of LV filling pressure and has been shown to predict cardiac events in HFpEF (96). However, in the most recent guidelines for the quantification of diastolic function, it is stated that optimal assessment of diastolic function cannot be made by any one measure and is best assessed by several echocardiographic parameters (97). Accordingly, it was recently shown that E/e′ did not accurately estimate LV filling pressure and neither did it identify increased LV filling pressure in patients with dyspnoea (98). As such, it appears that a multi-parameter approach to the assessment of diastolic function in HFpEF is needed (97). The parameters recommended for this purpose are mitral E/A ratio, E/e′, LA volume indexed to body surface area and tricuspid regurgitant velocity (97).

As previously discussed, LA structure and function is a sensitive barometer of LV filling pressure. Chronic exposure of the LA to elevated LV filling pressure causes LA dilatation (99). LA dilation is found in about half of all HFpEF patients (93). HFpEF patients rely more than HFrEF patients on atrial pump function to adequately fill their stiff and non-compliant LV. This puts great strain on the LA and, as a result, the prevalence of AF is high in HFpEF (approx. 40%) (100). When LA function ceases, LV filling in HFpEF becomes severely impaired and thus marks a stage of advanced disease. As such, AF in HFpEF is an independent predictor of mortality and hospitalization (100). Recent application of 2DS of the LA has resulted in the measurement of LA peak reservoir strain. This new parameter shows promise in categorizing diastolic dysfunction (101) and may have prognostic value in HFpEF (102). Nevertheless, the dependence of LA peak reservoir strain on LA size and LV longitudinal function should always be taken into account when assessing LA peak reservoir strain.

RV function is closely related to LV diastolic function, since high LV filling pressure will increase pulmonary pressures and cause greater RV afterload. Thus, pulmonary hypertension and RV dysfunction are highly prevalent in HFpEF (103). Pulmonary hypertension quantified by tricuspid regurgitation velocity is an independent predictor of mortality in HFpEF (103, 104). Furthermore, the presence of RV systolic dysfunction has incremental value in addition to the presence of pulmonary hypertension. RV systolic dysfunction may mark a stage in which the RV is no longer able to compensate for the increased afterload in HFpEF or it may be a marker of generalized cardiomyopathy affecting both the LV and the RV. Nevertheless, RV systolic dysfunction assessed by TAPSE is associated with AF and the comorbidity burden in HFpEF and is predictive of poor outcomes (105). The complicated geometry of the RV makes imaging challenging, but 2DS has recently been applied to the RV free wall and was shown to predict outcome in pulmonary hypertension (106). RV free wall strain may offer intriguing prognostic value in HFpEF. 

Once more, it becomes apparent that not one echocardiographic marker of cardiac structure or function is sufficient in HFpEF. A comprehensive examination is needed and results must be interpreted by the clinician on a personalized basis. We see that LV systolic and diastolic function, LA function and RV function offer prognostic value in HFpEF. As is stated in the current guidelines, the assessment of diastolic function is multifaceted and requires the assessment and interpretation of multiple echocardiographic indices (97). This is particularly true in HFpEF. Table 1 provides a list of studies that have identified echocardiographic prognostic parameters in HFpEF (Table 1).

### B-type natriuretic peptides and prognosis in HFpEF

BNP levels are lower in HFpEF than in HFrEF (64). This is likely due to lower LV wall stress in HFpEF compared to HFrEF, since the increased wall thickness and the reduced LV radius both decrease LV wall stress in HFpEF (Fig. 1). Despite the lower levels of BNP observed in HFpEF, the usability to predict all-cause mortality appears to be similar to HFrEF (64). BNP levels predict death due to worsening of HF, HF hospitalization and sudden death in HFpEF (107). Changes in BNP levels have also displayed prognostic value in HFpEF: In a study of 2612 HFpEF patients (the I-Preserve study), an increase in BNP levels over 6 months was associated with an increased risk of cardiovascular death and HF hospitalization, while a decrease in BNP levels at 6 months was associated with a trend towards a decreased risk of cardiovascular death and HF hospitalization. BNP levels may therefore become a valuable tool for guiding management and treatment in HFpEF patients.

## Echocardiographic risk prediction models in HF

It is now apparent that many echocardiographic markers hold prognostic value in HF. However, single measures of risk are rarely sufficient for the accurate estimation of prognosis in complex diseases such as HF (108). The topic of prognosis is essential to medicine and lays the foundation for clinical decision making. Accurate risk stratification allows clear communication of realistic expectations to patients and families and is instrumental in guiding evidence- and device-based therapies (22). Therefore, risk prediction in HF might benefit from the development of simple risk prediction schemes similar to the Systematic Coronary Risk Evaluation (SCORE) risk chart (109), and other prediction models currently used to estimate the risk of future cardiovascular disease in the general population. The estimation of risk from multivariable prediction models built upon the numerous established single-marker studies may offer more clinical value than simply identifying new single markers of risk (110, 111, 112). Many prediction models, with great heterogeneity in the number and types of predictors utilized, exist for HF; however, none have been deemed satisfactory (4, 110). Building a risk prediction model based on the accumulated data regarding echocardiographic predictors of risk in HF is cost-effective and feasible and may therefore represent a high-gain field of study in comparison to identifying, yet another prognostic marker. Echocardiography may be an optimal tool for personalizing risk stratification of HF patients and such efforts may help to maximize clinical applicability of the echocardiographic prognostic markers identified thus far. 

## Conclusion

B-type natriuretic peptides are useful in the exclusion of suspected HF; however, caution is warranted in the morbidly obese suspected of HFpEF. Echocardiography remains an essential procedure in HF. Echocardiography allows for accurate diagnosis and prognosis in both HFrEF and also in HFpEF. Future research should focus on combining echocardiographic prognostic markers into easily applicable prediction models in order to aid clinical decision making. 

## Declaration of interest

The authors declare that there is no conflict of interest that could be perceived as prejudicing the impartiality of this review.

## Funding

Daniel Modin was supported by a scholarship from the Medical Society in Copenhagen during the preparation of this manuscript. Tor Biering-Sørensen was supported by the Fondsbørsvekselerer Henry Hansen og Hustrus Hovedlegat 2016. The sponsors had no role in the study design, data collection, data analysis, data interpretation or writing of the article.
